# Agenda Setting in Outpatient Consultation of Older Adults With Long‐COVID

**DOI:** 10.1111/hex.70101

**Published:** 2024-11-24

**Authors:** Hao Zhao, Shuai Zhang, Wen Ma

**Affiliations:** ^1^ School of Foreign Languages and Literature Shandong University Jinan China; ^2^ School of Foreign Languages University of Jinan Jinan China

**Keywords:** agenda setting, conversation analysis, doctor–patient interaction, long‐COVID, older adults

## Abstract

**Introduction:**

Agenda setting in doctor–patient interaction refers to the process when various agendas are presented and responded to. This study revealed how Long‐COVID (LC) agenda items are managed by older adults and doctors during outpatient consultations.

**Methods:**

Based on audio recordings, we adopted Conversation Analysis (CA) to unveil under what circumstances the patient or the doctor set the agenda and how they collaboratively work to develop an LC topic. Data was transcribed in accordance with the transcription conventions developed by Jefferson.

**Results:**

Agendas were divided into three categories, namely primary, additional, and unmet agenda items. LC agendas were identified with specific characteristics based on qualitative analysis and older adults tended to seek more medical assistance, particularly concerning their chronic diseases. We observed that patients initiate agendas more often than doctors and it could happen at any stage of the visit, both parties can resist expanding the agenda, mostly in an indirect way, and agendas that contain more information with simpler turn‐constructions are more likely to be well‐addressed.

**Conclusions:**

Better quality of consultation was found when doctors sensitively detect the emotional change and the potential issues of the patients when they prevaricate to avoid the relevance of the previous infection. The findings also suggested that understanding the dynamics of agenda setting in consultations could lead to improvements in medical visit outcomes.

**Patient and Public Contribution:**

Patients and doctors in the study were colleagues in a university hospital. They were involved in various stages of this study to ensure it addresses real‐world concerns and improves healthcare outcomes. Throughout data collection, patients contributed by allowing their consultations to be recorded and providing feedback on their experiences. Findings were discussed with a patient advisory group to ensure the interpretations aligned with patient perspectives. Doctors were also actively engaged in disseminating the results through later consultations, ensuring broad accessibility and practical application of the research outcomes.

## 1 Introduction

The term *agenda setting* is used in doctor–patient interaction to describe the process of patients talking about their concerns, wishes, requests or goals, or clinicians raising subjects they consider important [1]. Alternatively, as Marvel et al. [2] explain, patients and healthcare providers can both establish the topics or issues to be discussed during the visit and either party can initiate, engage in, and switch the topic in later conversations, which may involve a collaborative work of prioritizing and negotiating [3, 4]. Agenda setting is a crucial aspect of medical communication, as it ensures that the most important issues are addressed and that both parties can communicate effectively [5]. The three main topics involved in primary care can be efficiently carried out if the agenda has been set collaboratively, which includes the way patients present problems, doctors propose questions and doctors diagnose and present treatment recommendations [6]. Specifically, from the physician's perspective, they may use open‐ended questions or other techniques to set agendas and encourage patients to share their complaints [7]. Hood‐Medland [8] further divides the agenda‐setting process into *agenda eliciting* and *reframing*, respectively referring to ‘physicians request from patients an upfront list of their medical concerns’ and ‘physicians reformulate the patient's talk at the beginning of the visit into an explicit agenda’. From the patient's perspective, she also uses *patient launch* to identify how they spontaneously initiate the conversation by presenting their issues, including physical symptoms, emotional or psychological concerns, questions about medications or treatments, etc. To cover these patient‐initiated agenda‐setting processes, modern healthcare has shifted towards a more patient‐centred approach where the patient's agenda is highlighted. A well‐processed agenda setting involves collaborative work by both parties until they reach a mutual understanding of the patient's concerns, priorities and expectations for the visit. It allows the physician to request more information [2], including addressing various issues such as follow‐ups on previous treatments or tests, preventive care and lifestyle change recommendations [9], leading to better overall consultation outcomes [10]. When agenda‐setting breaks down, the consultation process may be unscheduled or even chaotic, leaving serious issues unsolved, low satisfaction with the visit and patient resistance. These problems are exemplified especially during the post‐pandemic time when long COVID (LC) became a widespread concern.

While previous studies have explored doctor–patient interactions in general outpatient settings, few have focused on the specific communication needs and challenges of particular health conditions, such as LC. The U.S. Department of Health and Human Services reviewed nearly two million medical records of Corona Virus Disease 2019 (COVID‐19) and found that approximately one‐fifth of the recovered patients showed symptoms of LC (Section 4, note 1) [11]. It refers to the phenomenon where some individuals who recovered from the acute phase of COVID‐19 continue to experience symptoms for a prolonged period. WHO defines symptoms lasting more than 2 months after initial infection as post‐COVID conditions. The risk of post‐COVID‐19 syndrome was found to increase significantly with age [12], with 9.9% in individuals aged 18–48 years and 21.9% in individuals aged over 70 [13]. The 65+ demographic has a higher rate of multiorgan dysfunction that requires comprehensive techniques to present complaints [14]. Thus, setting the agenda for medical visits is a fundamental process for patients with severe or long‐term conditions to receive proper treatment and for doctors to highlight potential complications after an acute infection.

Although agenda setting is often used at the beginning of a clinical encounter, it can be processed at any stage as well [1]. Halkowski [15] introduces the concept of ‘agenda item’, referring to the concerns brought up by patients. Whether eliciting the problem at the beginning or the middle of the conversation, it is essential to verify the health issues presented are indeed LC conditions before the conversation moves forward because many assumed LC symptoms such as fatigue and hypertension may have other causes. During problem presentation, patients may share their own theories about their symptoms, which can inform the diagnosis but also influence the course of medical inquiry [16, 17]. This action is termed by Gill [18] as a *speculative explanation*. In the context of LC treatment, the doctor is therefore expected to identify whether the problem is associated with the experience of previous COVID‐19 infection. Normally, this is carried out by question–answer sequences or other forms of information exchanges to mitigate the epistemic disparity [19] and reach mutual understanding. Effective communication is strongly associated with a content health outcome and the satisfaction of the patient [20, 21], so if the agenda is shifted by one party, the other party may find ways to reframe it and carry it out again.

Conversation analysis (CA) is a method to learn the mechanics of conversation critically and scientifically, including verbal and nonverbal features, using video or audio‐recorded naturally occurring interactions [22]. CA emphasizes the sequences of communicative exchanges, illustrating how one utterance creates expectations for specific responses [23]. The study of doctor–patient interactions can greatly benefit from the application of CA. In medical settings, doctors and patients engage in highly structured and complex conversations that involve a range of communicative practices, such as presenting problems and providing treatment recommendations [24]. By analysing these interactions, researchers can identify communication breakdowns, misunderstandings and other challenges that may hinder effective healthcare communication. With the employment of CA, this research selects the most representative six examples to explain the questions: ‘How do Chinese senior patients and doctors set different types of Long COVID agendas in outpatient consultations?’ and ‘In which cases can they fully address these agendas?’ Based on the findings, we summarize the ways that healthcare professionals and patients articulate and respond to agenda items at various stages of the consultation process and provide suggestions for older adults consulting LC problems.

## 2 Data and Method

Participants in this research are general practitioners from a university hospital and medical visitors aged 65+ (Section 4, note 2), with no gender preference. Most of them are university staff or their relatives who actively participated in the study with verbal consent. Their requirements are mainly buying OTCs or taking daily medicines for themselves and/or for their family members. University hospitals are particularly essential for those who have chronic diseases with medication needs on a regular basis. Names and other personal information of the participants are replaced by pseudonyms to protect confidentiality. Given the period following the first wave of COVID, most patients consulted for health issues related to the pandemic, so we did not encounter significant difficulties in identifying elderly individuals suspected of having LC.

Naturally occurring conversations between doctors and visitors are collected as the source data. The audio data is recorded with a voice recorder (*Model: AMOI E20*) from January to February 2023, 6–10 weeks after the first wave of COVID‐19 infection involving 4 doctors (3 males, 1 female) and 70 patients. The total length is 395 min. The data is transcribed in accordance with the transcription conventions developed by Jefferson [25] manually, with the first draft completed by the first author and revised during the four 30‐min data sessions held by the three authors. In the transcriptions, ‘P’ stands for ‘patient’, ‘D’ for ‘doctor’, and ‘F’ for ‘family member’. The first line is the text written in Chinese phonetic alphabets, *pinyin*, and the second line is the translation in English.

### 2.1 Analysis and Findings

In this section, we illustrate the sequence organization of setting processes by senior patients and doctors when LC is the primary, additional and unmet agenda item.

### 2.2 Primary Agenda Item

#### 2.2.1 Patient Complaining About LC Problem at the Beginning of the Consultation

Normally, agenda‐setting process is initiated by doctors asking questions such as ‘What can I do for you today?’ and ‘How are you feeling?’ [26]. But since the collected data are from a university hospital, where the visitors are mostly acquaintances or co‐workers with the doctors, who are familiar with the visitors' health records, most of them skip the procedure of asking general questions. Typically, the conversation begins with the patient complaining about health issues persisting for more than 2 months after COVID‐19 infection.


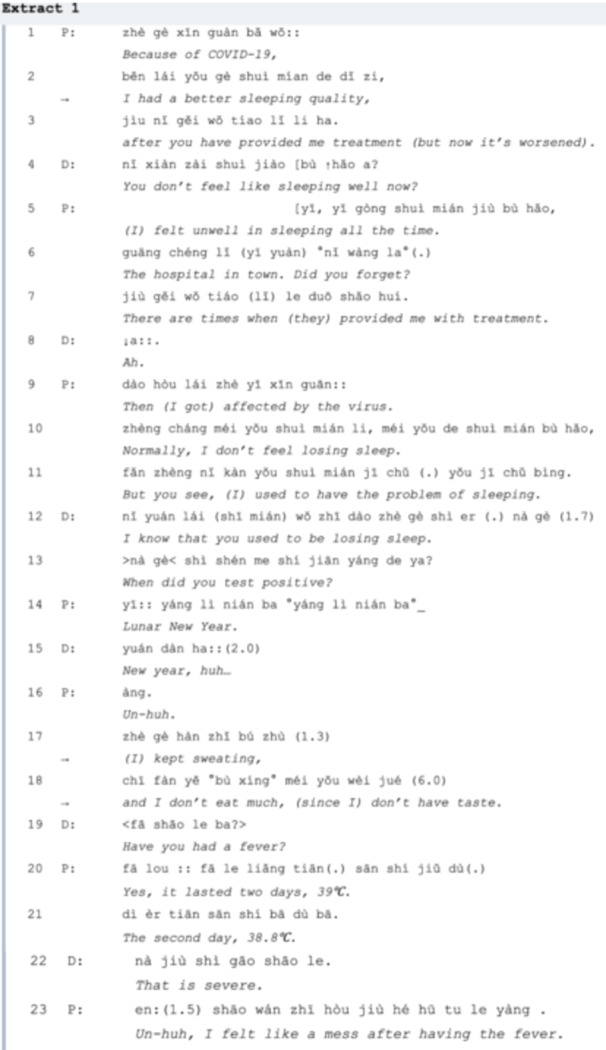


In Extract 1, to establish the agenda of this visit, the patient starts the conversation not by straightforwardly saying, ‘I want to discuss the problem of insomnia after COVID’, but by complaining about her problem of insomnia (*lines 01‐03*) after COVID‐19 infection over a month ago. So the doctor formulates the question ‘You don't feel like sleeping well now?’ in *line 04* to identify the primary agenda item of the patient in this consultation. In this turn and the rest of the conversation, the doctor responds to the patient actively because she already knows the information according to the patient's health records, as when the patient says ‘after you have provided me treatment' in *line 03* and her confirmation ‘I know’ in *line 12*. Even though the doctor is not always in a relatively unknowing (or K‐) stance compared to the respondent [27], the question in *line 04* is proposed to encourage the patient to provide more relevant information. However, the patient is talkative, which results in repetition and deviation. The patient then refers to her history of sleep loss and her previous visits to the hospital in *lines 05‐07*, but the information about her infection, what the doctor wishes to acquire, is not mentioned so far. Thus, in *line 13*, the doctor directly asks the patient for the exact date of their COVID‐19 infection. To answer this question, the patient uses an irregular way of referring to the date ‘sola calendar year’ in *line 14*, which acts as a trouble‐source turn [28], so the doctor repairs it as ‘New Year's Day’ with a modal particle ‘ha’ and a short extension in tone in seeking for confirmation. Without fully addressing losing sleep, the patient starts to talk about other problems (*lines 17‐18)*: sweating, loss of appetite and loss of taste, but the doctor focuses on collecting more information on the potential cause of these problems, which is her previous COVID‐19 infection. Two of the three questions the doctor proposed (*lines 04* and *19*) in this extract are basic *yes/no* questions, with different preferences, yet the patient provides excessive information, which results in other‐repair [22]. In *line 22*, the assessment ‘That is severe’. serves as sequence‐closing third (ibid.) and then the patient expands the sequence by describing her experience after the fever.

#### 2.2.2 Doctor Confirming LC Problem at the Beginning of the Consultation

When patients ask about a particular syndrome, the doctor may request more information. For example, in this case, the doctor uses *yes/no* questions to ask the patient whether she is referring to the condition caused by COVID.


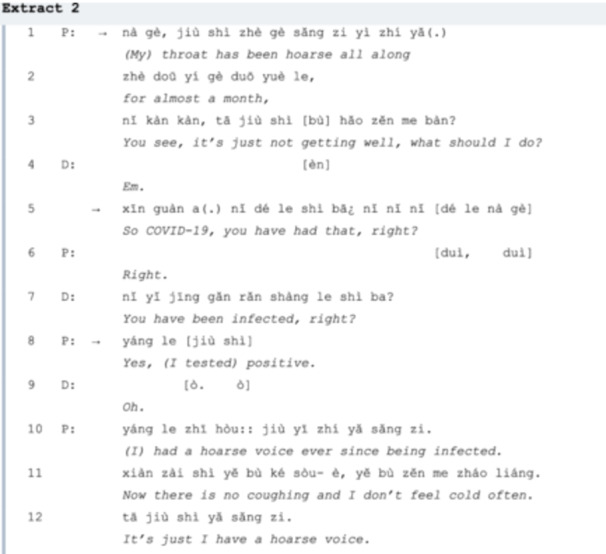


In Extract 2, the patient wishes to discuss her prolonged hoarse voice with the doctor. In *line 01*, the patient initiates the conversation with a short description of the problem, then in *line 03*, she immediately requests the doctor to pay attention, ‘you see’, followed up by a ‘what’ question. Since the patient did not mention in what condition she feels uncomfortable, the doctor has to affirm if she is talking about LC (*line 05*) with another question to make sure. Instead of following with an SPP, the doctor cancels the relevance of the First Pair Part (FPP) directed to him by replacing it with a new FPP. Here the preference of the question organization is *yes* by adopting the structure ‘statement+ straight interrogation’ [27], the doctor is expecting an affirmative answer. After reaching a consensus on the LC agenda, the patient describes her ‘hoarse voice’ in detail with two phrases ‘just’ and ‘always’ at the beginning and the end of the turn‐construction unit (TCU) in *line 10*, highlighting this symptom by mentioning ‘no coughing‘ and ‘don't feel cold often’. The doctor, in return, provides her with an explanation of the possible cause (*lines 13‐14*). Then she asks for the doctor's advice about whether she can take a particular medicine in *line 15*. In response, the doctor makes an affirmative announcement [29] and offers some casual healthcare products (e.g. Vitamin C).

### 2.3 Additional Agenda Item

#### 2.3.1 Patient Consulting Medication Due to LC in the Course of the Visit

Adhering to the treatment plan is critical for successfully handling the patient's illness [30]. One of the most common problems during the post‐pandemic age is medication adjustment (Section 4, note 3). Patients with chronic conditions such as high blood pressure and diabetes are required to maintain long‐term medication. However, due to persistent symptoms following an infection, many patients find it challenging to adhere to their previous routines.


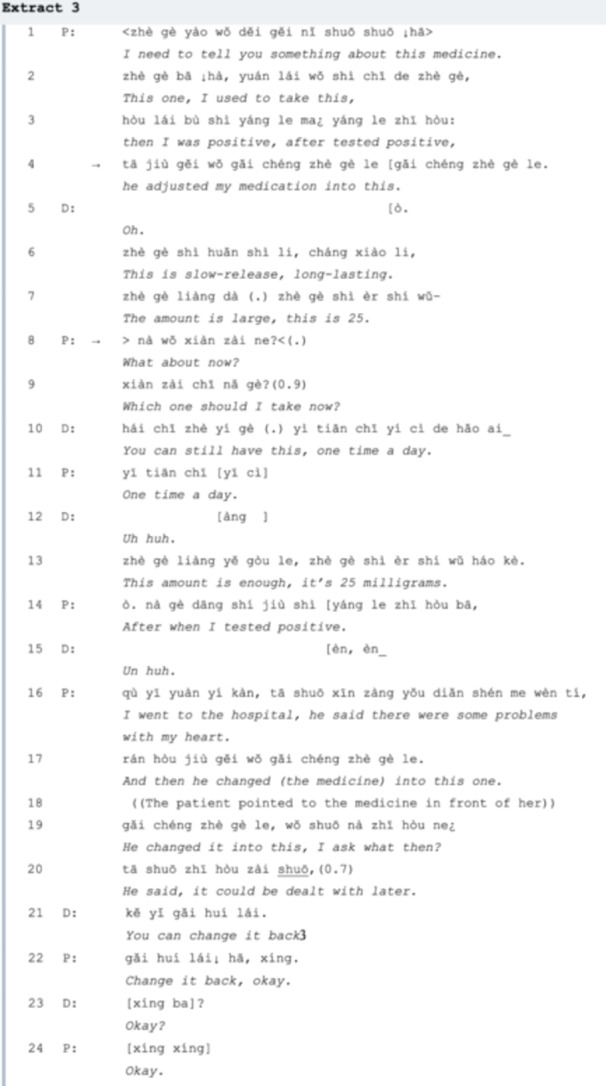


At the beginning of Extract 3, the patient experiences anxiety, but it is quickly alleviated upon being granted permission to resume her previous medication. Her utterance slowed with a serious tone to raise the doctor's attention in *line 01* and spontaneously explains her medication adjustment to him. The doctor introduces the new medication, but the patient interrupts, asking if she can switch back to her previous medicine. Instead of providing advice for the patient, the doctor illustrates the characteristics of the medicine. Consequently, in *line 08*. She hurriedly asks the doctor, ‘What about now?’ seeking advice. Immediately realizing her question lacks specificity, she quickly reformulates it to, ‘Which one should I take now?’ (*line 09*). Here, the agenda of medication adjustment due to the changing physical condition of LC has been settled, so in *line 10*, the doctor offers his recommendation of keeping the current new medicine and then goes on to discuss the course of it. But as *line 14* and *lines 16‐20* show, the patient withholds the response of acceptance [31] or defers the SPP by further explaining why she changed the medicine. With little time gap between the adjacent pairs, the doctor accepts her stance and agrees to change the original medicine back. In response, the patient repeats the doctor's words ‘change it back’ and says ‘okay’. In the closing section (*lines 23‐24*), the doctor re‐invocates the final decision that he and the patient made together with a closed question [32].

Additionally, most patients raise their problems by complaining about how they feel unwell and how the pandemic affects their lives. They describe their conditions and design the turn breaks for the doctor to point out the problem. In this way, doctors are expected to diagnose and perform treatment, although sometimes they may cancel the FPP and shift the topic.


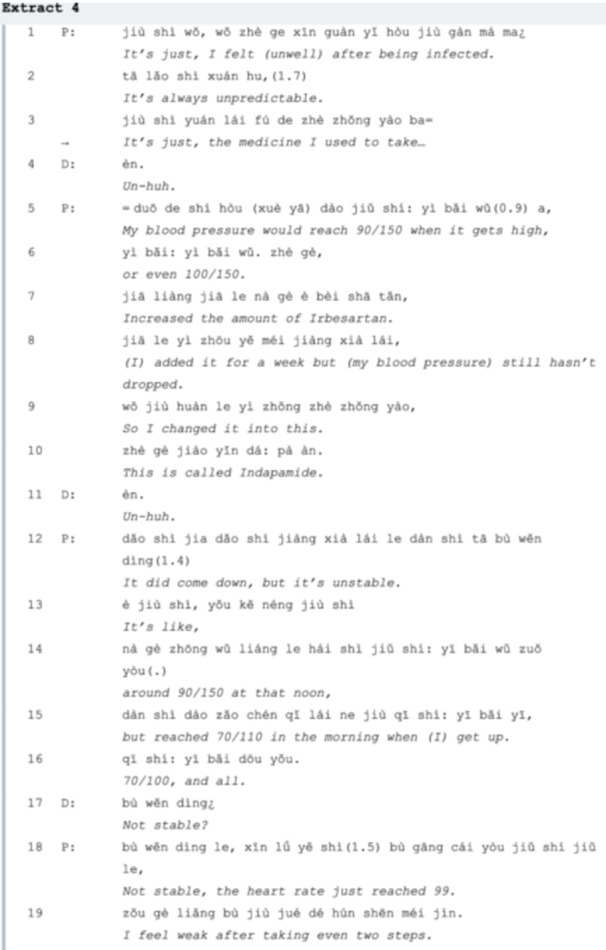



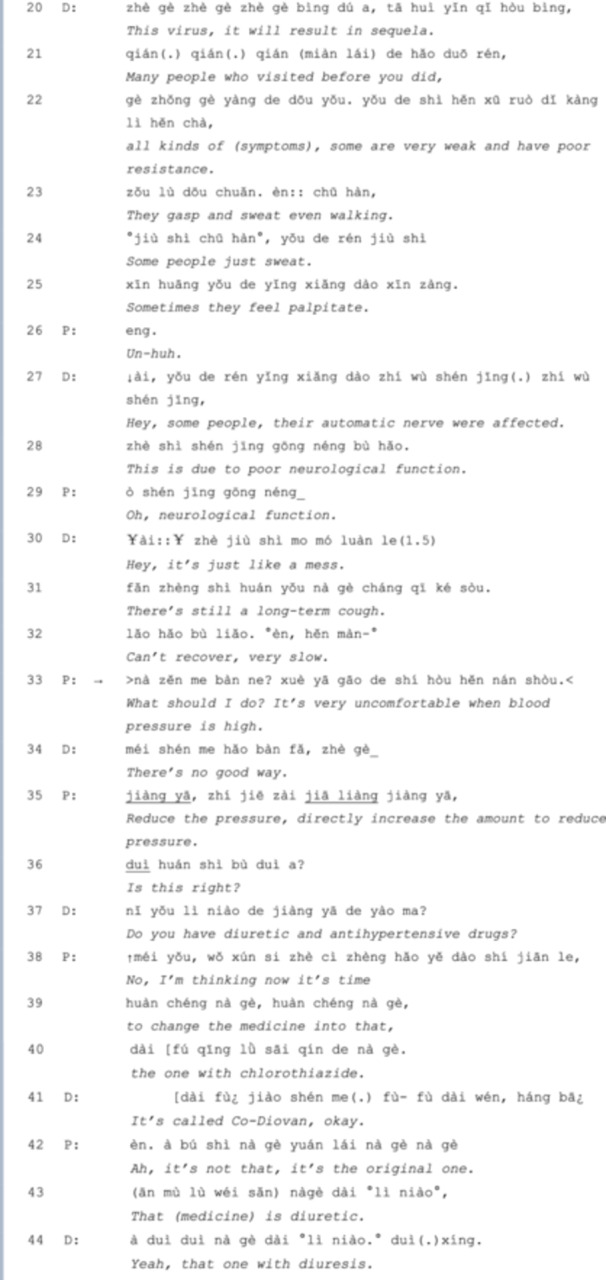


In Extract 4, the conversation is initiated by the patient complaining about his unstable blood pressure and heart rate using an assessment ‘it's always unpredictable’ (*line 02*), which also implies that he will take multiple turns to provide more explanation of how he changed his medicine due to the infection and how these changes were ineffective until *line 16*. The sequence organization here is similar to story‐telling [33], with the two major characteristics—pre‐expansion (*line 02*) and long FPPs (*lines 05‐16*) easily identified. This also indicates that the sequence of organization in outpatient consultations at university hospitals differs from that in general clinics. Then the doctor responds with another explanation of the possible causes and lists some other symptoms (*lines 20‐32*). Since the doctor did not elicit his primary concern, in *line 33*, the patient increases the speed of the utterance to raise the doctor's attention, using a *what‐* question ‘What should I do’ with another complaint: ‘It's very uncomfortable when blood pressure is high’, but these actions did not lead to a satisfactory answer. Therefore, in *line 36*, the patient uses a *yes/no* question to directly interrogate the doctor about whether he should increase the dose. The agenda of medication adjustment is now settled, but the doctor responds with another question, which shifts the topic from the dose to other kinds of medicine (*line 37*), so the patient uses a declarative tone with uncertainty (*lines 38‐40*). The doctor then cancels the initial FPP and proposes a new one, engaging in negotiations with the patient regarding a specific medication for treating hypertension. However, the patient misinterprets the doctor's intentions and initially resists the proposal in *line 42*, suggesting a medication he is more familiar with. Ultimately, they reach a consensus on a new medication in *line 44*.

#### 2.3.2 Doctor Concerning Health Issues Infected by LC in Follow‐Up Visit

As mentioned earlier, the consultation procedure in the recorded data differs from that of general clinics, where doctor eliciting agenda may be skipped. But in the following extract, the doctor is concerned about the patient's recent health status after the infection, so she elicits the LC agenda in the middle of this follow‐up visit.


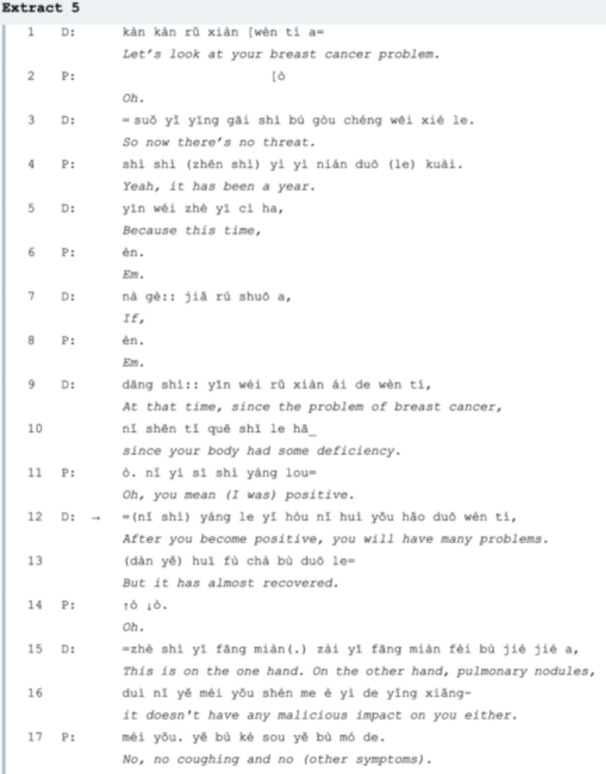



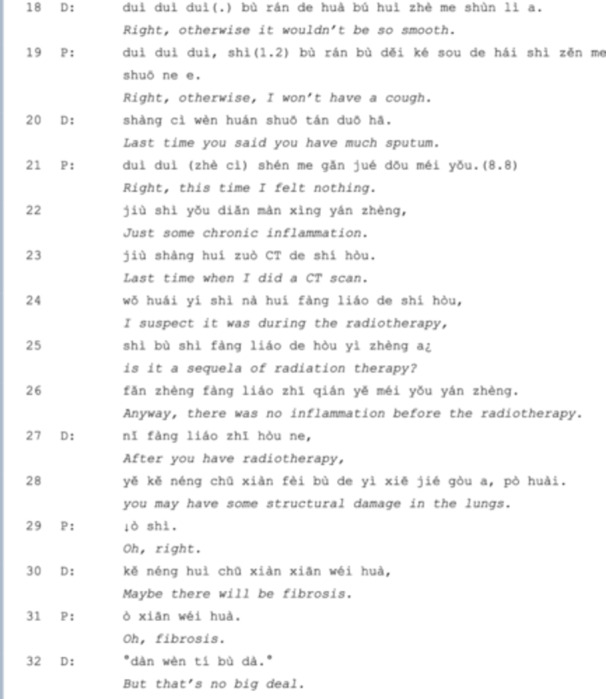


Extract 5 is a case of a patient with breast cancer in the third stage of chemotherapy after an operation. The enduring LC symptoms mentioned in her latest visit are now relieved, but the doctor is still prudent. In *line 12*, she points out that it is still possible for the patient to have persistent LC symptoms due to her physical deficiency (indicating the operation). After assuring the patient no longer has any symptoms (*line 15*), the doctor closes the conversation with an agreement of *line 18* by repeating ‘right’ three times and also reclaims the positive treatment outcome of the patient's pulmonary nodule, which is followed by the patient collaboration [22] of closing the sequence, repeating ‘right‘ three times (*line 19*). However, the conversation did not end. In *line 20*, the doctor initiates another TCU, referring to the patient's previous health record, which is immediately agreed upon by her (*line 20*). After an 8‐second gap, the patient self‐speculated that her inflammation could be the result of radiotherapy in *lines 23‐27*. Instead of answering the *yes/no* question proposed by the patient in *line 26*, the doctor affirms the speculation with an explanation (*lines 28‐29* and *line 31*).

### 2.4 Unmet Agenda Item

Not all agendas are addressed with efforts made by both the patient and the doctor. Doctors are required to help the patient prioritize their concerns and reframe them in a way that is consistent with the goals of the visit. This can involve identifying the most pressing concerns, helping the patient to clarify their goals and making sure to address the most important issues. When the patient brings up a problem in the end stage of the consultation, it may not be fully extended due to time limits and other reasons. In this case, the agenda‐setting process is not complete and finishes immediately after initiation, resulting in an ‘unmet concern’ [34].


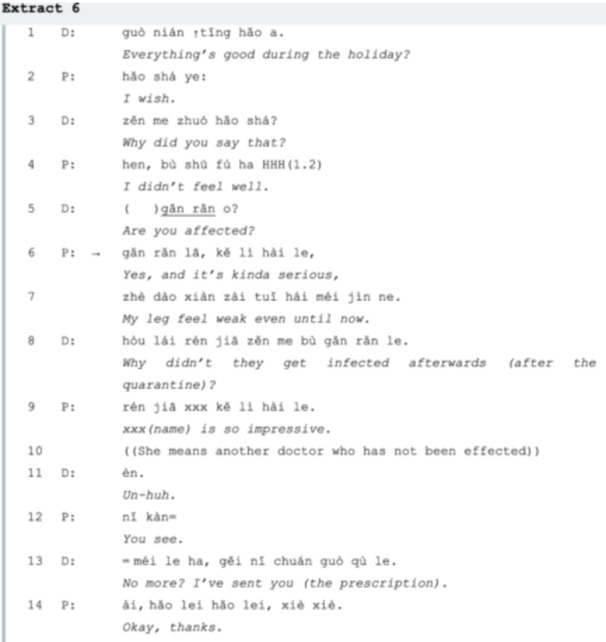


In *line 01* (Extract 6), the doctor starts a casual conversation with a patient by a greeting ‘Everything's good?’, a question with a preferred *yes‐good* answer, but unexpectedly, the patient responds with a complaint ‘I wish’. (*line 02*). So the doctor reformulates it into a ‘why’ question in *line 03* to ask what happened. Instead of providing an answer to what happened, the patient gives a vague answer in *line 04*, ‘not comfortable’. The doctor presupposes the patient is talking about the pandemic, so he initiates a new adjacency pair with the question in *line 05*, ‘(you have been) infected?’, the SPP is the confirmed answer in *line 06*. Then the patient expands the sequence with an assessment ‘serious’ and elicits her LC problem. But she does not seek medical assistance or advice from the doctor, instead, she closes the conversation and pauses setting her agenda of leg fatigue. The doctor agrees on the closure of this sequence and shifts the topic to gossiping about their acquaintance in *line 08*.

### 2.5 Findings

Based on the analysis above, we find that older adults typically raise an average of two health issues during each consultation and the characteristics of the LC‐related ones are detailed in Table 1.

**Table 1:** Characteristics of the LC agenda.

	Characteristics of the LC Agenda
**Formation**	Lack of pre‐expansion: Typically begins without any preliminary discussions. Terminology variations: Patients/doctors refer to the COVID‐19 infection using ‘positive’ or ‘it’, indicating variability in how LC is framed. Reduced duration for patient input: doctors allow less time for patients to express their LC‐related concerns compared to regular consultations.
**Initiation**	Primarily Patient‐Initiated: Mostly initiated by patients, occasionally by doctors. Multi‐Step Process: Often involves various actions, such as requests, complaints, or explanations. COVID History Not Always Referenced: When the patient raises the LC issue, their prior COVID infection may not be mentioned at the outset.
**Response**	Indirect Rejection: If a participant chooses not to engage with the agenda, they tend to shift, reframe, or ignore the topic rather than reject it outright. Patient‐Initiated Suggestions: When dissatisfied with the doctor's treatment recommendations, patients may propose their own medication suggestions for the doctor's approval.

In Extract 2, it is suggested that a ‘hoarse throat’ is not initially linked to the patient's infection history, which requires the doctor to probe for clarity. Additionally, during follow‐up visits concerning sensitive health issues like cancer (Extract 5), it is crucial for doctors to approach the potential impact of COVID with care, since the emotions are not always verbalized [35]. Besides, to prevent significant issues from becoming unmet agenda items, doctors should actively seek out potential concerns such as ‘weak leg’ in Extract 6A, similar result is shown in Cohen et al.'s study, [36] confirming “an excess risk for persistent and new sequelae in adults aged ≥ 65 years” after SARS‐CoV‐2 infection (*line 7*,). Sharkiya [37] concludes that verbal/non‐verbal strategies affect the outcomes of 65+ patient‐centred care, and more non‐verbal communication such as gesticulation and visual contact may avoid the ignorance of potential health problems [38, 39].

It would be advantageous for both parties if the visitor lists the issues in advance, as some may be overlooked if mentioned spontaneously. For example, in Extract 1, various LC symptoms (e.g., sweating, loss of appetite) are noted before the primary concern of insomnia (*lines 1‐12*) is fully addressed. In Extracts 2 and 3, detailed information about COVID‐19 infections and cardiac issues should be communicated in a more structured and timely manner, enabling doctors to offer appropriate medication suggestions efficiently. Furthermore, senior patients who are familiar with their medications may benefit from proposing their own treatment plans (Extract 4), though casual conversation can sometimes lead to the neglect of critical concerns (Extract 6). We sum up the suggestions for fully addressing LC agendas as listed in Table 2:

**Table 2:** Suggestions for setting agenda in outpatient consultations.

Doctor agenda setting	Older visitor agenda‐setting
Patients' speculative interpretation require doctors to determine whether the problem is related to the COVID‐19 infection experience by obtaining more details. Be cautious in language use, promptly detecting and alleviating patients' negative emotions by using authoritative and professional knowledge to make them feel emotionally supported. Be sensitive to the patient's issues. Guide the patient to articulate potential problems using both verbal and non‐verbal actions.	Consider preparing a clear list of concerns to be addressed at the session in advance to avoid consuming too much time or leaving out important issues. Describe the medical or infection history (time and symptoms) in a more structured and explicit manner. Voluntary providing preferred treatment plans sometimes leads to better consultation outcomes. Minimizing casual chats may enhance consultation efficiency.

## 3 Discussion and Conclusion

Public health emergencies have been persistent challenges throughout human history, as epidemics of infectious diseases have repeatedly appeared. They significantly impacted individual health and broader society and economics, also triggering a range of physical and psychological conditions, especially in the vulnerable senior population. This article explains the sequence organization of agenda setting through six representative examples of elderly individuals inquiring about long‐term post‐COVID symptoms and compares the presentation of agenda items by patients or doctors at various stages of the consultation. Byrne and Long [40] conclude that patients typically raise an average of three health concerns, with the first being the least important, while Callahan (2000) found that older adults tend to have longer consultation times but usually address fewer agenda items. In our data, we find that most participants bring up two agendas. Patients seeking medication suggestions for chronic conditions would focus on the topic of how LC negatively affected their daily lives. However, two patients are also found to come up with more than three topics including multiple LC issues. Beckman et al. [41] find that physicians would leave an average of 18 s before interrupting patients for presenting their concerns, but we observe that doctors allocate less time for patients to discuss their issues, either because many patients present similar problems, leading doctors to assume a shared agenda, or due to their familiarity with patients' medical histories.

Describing symptoms that are suspected to be LC is the core phase of the whole visit and this is usually carried out by explanations. For most first‐time visits, history‐taking can be an essential procedure to mitigate the epistemic gap between the two speakers [19, 42]. By constructing complete accounts of the problems, the doctor can therefore perform their professional skills in diagnosis and treatment [43]. At the initial stage of the consultation, a patient may complain about a persistent symptom, making it the prior issue to be discussed. In Extract 1, it only takes two turns for insomnia confirmed as the main agenda item, indicating relatively high efficiency. This process differs from Heritage's [27] study, where doctors ask ‘What brings you here today?’ to elicit the main concerns of patients. There are two possible reasons: first, the conversations occurred at a university hospital where visitors are often acquainted with the doctors, who know their health records; second, the recording time corresponds to when most visitors consult about LC‐related issues. In comparison, the patient in Extract 2 talks about a persistent hoarse throat at the beginning of the consultation but does not connect this symptom to her previous experience with COVID. The doctor is then forced to inquire about the patient's infection history, which takes six turns, displaying lower efficiency.

Treatment recommendation, especially in a situation like medication adjustment, can be seen as ‘a bilateral process in which the physician proposes but the patient disposes of’ [6]. Medication adjustments due to COVID‐19 infection typically become an additional agenda item. However, despite both conversations (Extracts 3 and 4) focusing on adjustments to chronic illness medication, their effectiveness varies. In Extract 4, the patient's narrative style regarding their main medical concerns is more akin to storytelling in CA, since it ‘involves recounting events that require more than one turn‐constructional unit to tell’ [44]. CA recognizes an adjacency pair as the unit for sequence construction and it is pair‐type related (question‐answer, invitation‐accept/decline comes always in pairs). That is, the recognizable production of an FPP is what triggers a complete adjacency pair, which forms a successful interaction [22]. Hence, communication efficiency will be hampered if one of the parties does not respond according to the pre‐supposed answers to questions such as producing a storytelling‐style discourse.

Clinicians can also raise subjects they consider essential [1]. The patient in Extract 5 had just gone through a radiotherapy and completed a breast cancer surgery and her short‐term self‐diagnosed ‘chronic inflammation’ of suspected ‘fibrosis’ was discussed in the previous visit. As the consultation continues, the patient identifies that the doctor's primary concern is the pulmonary nodules that emerged after her recovery from COVID‐19. The patient then raises this issue, leading to a mutual acknowledgement of the agenda concerning these lung complications. The conversation in this follow‐up visit progresses smoothly since both participants agree upon the agenda and the patient can directly point out the concern that the doctor intends to discuss.

An unmet agenda item may be brought up, especially at the end stage of the visit. The patient in Extract 6 does not wish to mention the condition of long‐term fatigue in the first place when the doctor initiates the chat. After the patient detects there is something unusual in the patient's health status, he uses a question to elicit more problem talk from the patient. But the doctor recognizes it as a complaint and does not provide much attention nor does the patient further extends, leading it to an unaddressed problem, or a ‘hidden agenda’ [45]. Therefore, doctors are expected to be more prudent in linguistic usage, timely detect underlying concerns, and try to alleviate negative sentiments by exploring potential health problems, performing their authority, and being empathetic to the patient, as a way to make them feel emotionally supported and aligned.

In conclusion, the contribution of this study can be mainly described from three aspects. Methodologically, it adopts CA to examine how agenda setting is achieved in outpatient consultations. By applying this rigorous method, this study provides detailed insights into how healthcare providers and patients negotiate and establish the topics to be discussed during the visits. Theoretically, it contributes to the understanding of agenda setting in healthcare interactions. While previous literature has been focused on the role of healthcare providers in setting the agenda, relatively little attention has been paid to the role of patients. This study addresses this gap by examining how older Chinese individuals engage in agenda setting during outpatient consultations. Practically, it attends to the urgent need to provide suggestions for improving the satisfaction and engagement of older adults in their medical care, particularly regarding LC. Nevertheless, limitations exist since the processes of outpatient consultation in school hospitals are often simplified, resulting in ignorance of some concerns due to overlapping consultation times. Besides, the close relationship between doctors and visitors can make their interactions more personal and less institutional, higher lifestyle discussion and rapport‐building rates may relate to adverse outcomes while seeking and giving sufficient information results in satisfactory ones [46]. Finally, the limited data could be raising a concern of generalization, so future research should collect audio/video data from various departments of comprehensive hospitals to further support the findings.

## 4 Notes

1. Symptoms can be diverse individually, ranging from general symptoms like fatigue, fever and respiratory; heart symptoms like breathlessness, cough, chest pain and heart palpitations; neurological symptoms like brain fog, headache, insomnia, loss of taste and/or smell, depression or anxiety; digestive symptoms like diarrhoea, and many others. These symptoms may emerge after recovery and may fluctuate or recur over time, but the exact causes are still uncertain, even though it is believed to be associated with ongoing inflammation and autoimmune response.
2. Although the elderlies are defined as those who are older than 80 in some articles [47, 48], most studies target doctor–patient interaction and recognize 65 as the demarcation point [49], as well as in the present study.
3. Adjustment includes changing the dose, replacing the original medicines or temporally pausing intake.

## Author Contributions

**Hao Zhao:** writing–review & editing; writing–original draft; methodology; investigation; conceptualization; software; data curation; formal analysis; visualization; resources. **Shuai Zhang:** supervision; writing–review & editing; funding acquisition; conceptualization; validation. **Wen Ma:** supervision; funding acquisition; conceptualization; methodology; validation; project administration.

## Ethics Statement

This study was approved by the Ethics Committee of the School of Basic Medical Sciences, Shandong University (approval no. ECSBMSSDU2019‐1‐056).

## Consent

We confirm that all our participants gave informed verbal consent.

## Conflicts of Interest

The authors declare no conflicts of interest.

## Data Availability

The data that support the findings of this study are available from the corresponding author upon reasonable request.
